# Shades of grey: choice, control and capacity in alcohol-related brain damage

**DOI:** 10.1192/bjb.2023.92

**Published:** 2024-12

**Authors:** Nuala B. Kane, Norella Broderick, Emily Rao, Alex Ruck Keene, Rahul Tony Rao

**Affiliations:** 1 Health Service Executive, Dublin, Ireland; 2 University of California San Diego, La Jolla, California, USA; 3 King's College London, London, UK

**Keywords:** Consent and capacity, alcohol disorders, dementias/neurodegenerative diseases, ethics, psychiatry and law

## Abstract

Liaison psychiatrists have identified that conducting capacity assessments in general hospital patients with alcohol-related brain damage (ARBD) can be challenging. This educational article uses the fictitious case of a man with ARBD, alcohol dependence and significant self-neglect, focusing on assessment of his capacity to decide about moving into a care home on discharge. We provide an overview of clinical, legal and ethical literature relevant to decision-making and capacity assessment in individuals with ARBD, with the aim of guiding clinicians approaching complex capacity assessments.

## Clinical scenario

Mr X is a 52-year-old man with alcohol-related brain damage (ARBD) and active alcohol dependence. He is brought to the acute hospital in an unkempt, disoriented and inattentive state and is medically admitted and treated with chlordiazepoxide and high-dose parenteral thiamine. Mr X is known to be a frequent hospital attender with alcohol withdrawals and complications of alcoholic liver disease. He lives alone in a council flat, and there are increasing complaints from neighbours that the property is in a neglected state, with empty bottles, clutter and vermin. Prior to previous hospital discharges, Mr X agreed to a care package, but this plan consistently broke down owing to his failure to admit carers into his home. On this occasion, the multidisciplinary team (MDT) view is that discharge to residential care that restricts access to alcohol should be considered. Following completion of Mr X's alcohol detoxification, the team explains to him the serious medical and social complications of his alcohol use, the importance of abstaining from alcohol and the MDT recommendation. However, Mr X declines to consider a care home and insists that he wishes to return home and to continue drinking ‘in moderation’. Considering this a complex case, the social worker requests a liaison psychiatry opinion on whether Mr X has capacity to make decisions about his care and residence on discharge.

### Key questions

- How can ARBD and alcohol dependence affect decision-making ability?
- How should you approach assessment of capacity to consent to care and residence in this case?
- What guidance do clinical studies, legal cases and the ethics literature provide?

## Introduction

Liaison psychiatrists, often called to help with capacity assessments in the general hospital, have identified that assessments can be challenging in patients with ARBD who refuse care. In a recent qualitative interview study, they noted that good verbal skills in these individuals can hide significant cognitive and functional impairment, and executive dysfunction can be associated with a lack of ‘follow through’ on care plans made with healthcare professionals.^1^ Previous literature has raised further clinical and ethical considerations. For example, when is the right time to carry out a capacity assessment? Is coercive intervention justified when addiction is causing harm?^2,3^ This article gives an overview of literature and case law to guide clinicians approaching these assessments. We hope that the insights provided might also be applied to other complex capacity situations. The legal framework discussed is the Mental Capacity Act 2005 (MCA) of England and Wales; the MCA defines incapacity (which must be caused by an impairment of mind or brain) as the inability to understand, retain, use or weigh relevant information, or to communicate the decision.

## Review of the literature

### Characteristics of ARBD

Alcohol-related brain damage is an umbrella term for chronic neuropsychological disorders caused by direct and indirect effects of alcohol on the brain,^4^ and usually understood to include both alcohol-related dementia and Wernicke–Korsakoff syndrome.^5^ Proposed pathophysiological mechanisms include the direct neurotoxicity of ethanol and its metabolite acetaldehyde, thiamine deficiency and vulnerability to hepatic encephalopathy, traumatic brain injury and cardiovascular risks.^4,6^ Structural and functional brain changes in ARBD affect the frontal lobes (prefrontal cortex), limbic system (hippocampus, hypothalamus and mamillary bodies) and cerebellum.^4,7^ Typical cognitive deficits include executive dysfunction, loss of episodic memory (prominent anterograde amnesia in Wernicke–Korsakoff syndrome) and visuospatial deficits, with relative sparing of language in comparison with Alzheimer's disease.^8^ In contrast to other dementias, people with alcohol-related dementia are more likely to be younger, male, socially isolated and have significant comorbidity.^8^ There is limited evidence for pharmacological treatment of ARBD^8–10^ but acute treatment of suspected Wernicke's encephalopathy with high-dose parental thiamine is advised to prevent chronic deficits.^4^ Stability or partial reversibility of clinical deficits and neuroradiological changes can be achieved with abstinence from alcohol.^4,8^ However, people with ARBD are less likely to move to abstinence than peers with alcohol dependence and intact cognition.^11^

### Alcohol and decision-making

The ‘competing neural systems account’ models alcohol dependence (and other addictions) as an imbalance between impulsive and reflective cognitive systems, leading to dysfunctional decision-making in relation to alcohol use. The overactive impulsive system involves strong associative memory between alcohol-related cues and ‘automatic’ behaviour (mobilising the dopaminergic system of the amygdala–striatal circuit), while the underactive reflective system involves executive functions (various regions of the frontal lobe and striatum) that become less able to regulate the impulsive system to achieve adapted behaviour.^7,12–14^ It has been suggested that greater impairment of executive functions (working memory and inhibition capacity) in ARBD increasingly predisposes to drinking behaviour dictated by the impulsive system.^7^ Beyond decision-making about drinking, studies evidence wider decision-making impairments in individuals with alcohol and other addictions. These include exaggerated delay discounting: compared with controls, dependent individuals significantly prefer less substantial, more immediate rewards over more substantial, delayed rewards.^14^ Risk valuation is also impaired: individuals with alcohol dependence tend to make riskier decisions^15–18^ as tested by the Iowa Gambling Task.^19^ Even those now abstinent from alcohol continue to perform more poorly than controls,^16^ suggesting persistent decision-making deficits.

### Clinical studies of decision-making capacity

Little is known about how these deficits correlate with real-world decision-making and decisional capacity. To date, no study has systematically investigated capacity for personal care decisions in individuals with ARBD or alcohol dependence. Findings from other patient groups and other decisional capacities may be relevant. A descriptive study found that liaison psychiatrists often determine patients with substance use disorders to lack capacity for healthcare decisions.^20^ Several studies have found people with Alzheimer's disease more likely to lack capacity for various decisions than unaffected peers, although with significant heterogeneity in those with mild to moderate cognitive impairment. Studies of individuals with traumatic brain injury showed that impaired capacity for treatment and financial decisions is prevalent and strongly correlated with severity of the injury.^21,22^ In both Alzheimer's and traumatic brain injury groups, memory and executive function deficits were predictive of decisional impairment.^23–26^ A qualitative study has suggested that failure to ‘use or weigh’ in brain-injured patients with frontal lobe syndrome may relate to difficulty integrating an abstract awareness of their deficits into real-world decision-making.^27^ In an interview study, liaison psychiatrists recommended that clinicians assessing capacity in patients with ARBD should carry out repeat assessments, incorporating evidence of the patient's real-world decision-making.^1^

### Relevant case-law

Although studies of incapacity in different clinical groups can be helpful in guiding one's approach to assessment,^28^ only a functional capacity assessment can determine an individual's capacity to take a specific decision at a specific time. The highest arbiter of capacity assessments under the MCA is the Court of Protection (CoP), which offers a useful data-set of published judicial decisions.^29^ To date, only two published judgments have expressly considered capacity in individuals with ARBD (Table 1). In both cases (perhaps surprisingly), the individual was found to have capacity for care and residence decisions. Clinical factors discussed included the individual's memory and planning abilities, insight into his (in)ability to control his drinking, ability to mask his cognitive deficits, and evidence of reversal of cognitive impairment with abstinence. Legal factors such as time and decision specificity were discussed; in one case, the judge cautioned that evidence about the individual's capacity to make decisions about alcohol was only relevant when tied directly to the ‘operational’ decision (capacity for care/residence).

**Table 1:** Published Court of Protection judgments on capacity in alcohol-related brain damage (ARBD)

Case	Impairment/issue	Background	Evidence for incapacity	Judge's determination
*London Borough of Tower Hamlets v PB* [2020] EWCOP 34	Capacity of PB, a 52-year-old man with ARBD and dissocial personality disorder, to make residence and care decisions	PB had a history of homelessness and recurrent hospital admissions with alcohol-related complications. At the time of the hearing, he was living in a residential care unit that placed restrictions on his access to alcohol; his wish was that these restrictions be lifted.	The expert witness argued that PB was unable to use or weigh information (most pertinently his own inability to moderate his drinking) and attributed this to both to his alcohol dependence and executive dysfunction associated with ARBD.	The judge deemed that PB had capacity to make the relevant decisions, arguing that the expert's test would have ‘the alarming effect of rendering most addicts incapacitous’. He noted that PB expressed an appreciation of the consequences of drinking to excess and an aspiration to moderate his drinking.
*X v A Local Authority & Anon* [2014] EWCOP 29	Capacity of X, a retired lawyer with Korsakoff's syndrome, to make decisions about his care, residence and medical treatment	After a lengthy admission under the Mental Health Act, X was transferred to a care home under deprivation of liberty safeguards, which he subsequently appealed.	X's social worker, who had known him throughout his illness, expressed concern about his unrealistic expectations of the future and his ability to mask his cognitive difficulties.	The judge concluded that ‘although he suffers from short term memory problems, he retains sufficient information to be able to deal with planning’. The judge was further persuaded by evidence of an independent psychiatrist (who saw X once), who emphasised that X's mental state appeared to have improved over recent months.

Interestingly, neither judgment gave much detail on the individual's cognitive impairment. This contrasts with other CoP judgments examining capacity in individuals with mixed dementias caused in part by alcohol use. For example, in *D v R & S* [2010] EWCOP 2405, the case of a man with dementia ‘most probably caused by alcohol use and cerebrovascular disease’, there was extensive reference to structured psychological measurement, including the Mini Mental State Examination (MMSE) and specific frontal lobe tests.^30^ The judge concluded that the individual's memory and frontal deficits caused an inability to understand and weigh relevant information.

In *London Borough of Tower Hamlets v PB* (Table 1) the judge expressed concern lest ‘most addicts’ be considered incapacitous. More broadly, two other CoP judgments considered capacity in the context of alcohol dependence and so are worth mentioning. In *An NHS Foundation Trust v Ms X* [2014] EWCOP 35, the experts contrasted a young woman's capacity to decide about alcohol use with her lack of capacity regarding treatment of her anorexia:^31^ ‘ … she appeared to be making choices about when to drink, when to drink more, and when to drink less’. In *RB v Brighton and Hove CC* [2014] EWCA Civ 561, the court distinguished between a man's decision-making ability before and after a traumatic brain injury affecting his frontal lobe; he had a prior history of alcohol dependence, criminality and homelessness. Expert evidence argued that an ‘ordinary alcoholic [ … ] does not have the frontal lobe damage which means that a person such as RB works on impulse [ … ] Alcoholics can weigh up their decisions’.

### Ethical debates on capacity in alcohol dependence

Ethical debates on capacity in substance use disorders frequently focus on whether dependent individuals have capacity to decide about use of their addictive agent and treatment for their addiction. Responding to the practice of prescribing heroin to people with heroin use disorder in research trials, Charland argues that people with addiction – by nature of their disease – have compromised ability to ‘weigh risks and benefits’ associated with heroin use and thus are incompetent to consent to its prescription. Invoking studies of altered neurocircuitry in people with addiction, he describes the transformative power of addiction-related ‘compulsion’. Under duress of such compulsion, he argues, people with addiction, regardless of their behavioural state, approach decisions with a fundamentally changed set of ‘pathological values’ that compromises capacity.^32^ Craigie & Davies compare ‘impaired control’ in alcohol dependence with that in anorexia nervosa, pointing out that anorexia is often cited to impair capacity regarding treatment.^31^ In contrast, Foddy & Savulescu reject the pathologised ‘disease view’ of addictive behaviour as a ‘fiction of affliction’. They argue that people with addictions remain fully autonomous, as addictive desires are essentially normal (if especially strong) desires towards pleasure, and only ‘irresistible’ forces render choices non-autonomous.^33^ Importantly, these ethical arguments do not, without additional reasoning, apply to the question of whether addiction impairs capacity to make other decisions, such as care or residence. However, where a care plan or residence environment restricts or forbids access to alcohol, they may become relevant. Further, a recent paper argues that executive dysfunction in people with opioid use disorder who refuse medical care after overdose may cause these individuals to be ‘unmoved’ by the risks of leaving hospital and may impair their capacity for this decision.^34^

## Reflections and considerations

Mr X has ARBD and alcohol dependence, with evidence of severe self-neglect and a history of refusing home supports that he has previously agreed to. Assessment of his capacity to consent to admission to a care home, where his access to alcohol is likely to be restricted, raises clinical, legal and ethical issues.

Although studies evidence brain changes and functional decision-making impairments in alcohol and other addictions, the CoP cases show that there is strong resistance in the courts to considering that an ‘ordinary alcoholic’ might lack capacity. The ethics literature is divided, but there is likely to be a pragmatic element at play here. Considering that persons with alcohol dependence may lack capacity to make decisions about alcohol raises the policy question of whether restrictive measures should be initiated in their best interests to promote abstinence. However, enabling recovery from addiction seems more likely to be achieved by empowering rather than diminishing a person's sense of agency.^35,36^ There is little evidence for efficacy of coercive treatment for dependence syndromes outside the criminal justice system^37^ and, with limited exceptions,^38,39^ there is little appetite for this practice worldwide. Whether or not directly reflecting this evidence, deprivation of liberty for substance dependence is not legally sanctioned under the MCA or the Mental Health Act 1983 (England and Wales).

Nonetheless, individuals with ARBD, such as Mr X, can have severe cognitive impairment and impaired functioning, which mirrors other dementia syndromes. Social isolation, poor engagement and relatively preserved verbal abilities allowing the masking of impairment might all contribute to under-recognition of ARBD and its consequences for decision-making capacity. Cognitively, Mr X may have deficits in episodic memory that prevent his recall of the neglected condition of his home or of events following previous hospital discharges. He may have executive dysfunction impairing his ability to reason about risks, to integrate abstract knowledge of his care needs into decision-making or to follow through on agreed care plans. Further, there is a complex interplay between the effects of ARBD and alcohol dependence on decision-making about drinking, and the impulsivity and disinhibition characteristic of executive dysfunction in ARBD is likely to interact with and amplify reinforcement-driven addictive behaviour. This may influence Mr X's ability to decide to stay in residential environments restricting his access to alcohol.

As clinicians confronted with alcohol-related presentations like that of Mr X, it is always worthwhile to reflect on the role of negative countertransference,^40^ wider stigma and societal value judgements on who is ‘to blame’ and who ‘deserves treatment’.^31^ Finally, it is worth noting that competing ethical and legal duties are at play in these complex cases. There is a fallacy in thinking solely about capacity (and, within that, capacity to make decisions about alcohol and care), as opposed to locating discussion within the scope of wider obligations to safeguard vulnerable persons,^41^ for instance under legislation giving effect to the European Convention on Human Rights, as well as duties under the Convention on the Rights of Persons with Disabilities.

### Practical management

1. Delay the capacity assessment until Mr X's acute medical problems are treated. Ensure that an adequate dose and duration of chlordiazepoxide has been given to treat alcohol withdrawal fully. Assess for features of Wernicke's encephalopathy (note that the full triad of oculomotor disturbance, ataxia and altered mental state is present in only 23% of cases) and if it is suspected, continue treatment with high-dose parenteral thiamine until no further clinical improvement is noted.^42^ Other causes of delirium, such as hepatic encephalopathy, brain injury or infection, should be identified and treated appropriately. Screen for and treat comorbid mental health conditions, including mood or psychotic disorders.
2. Carry out a thorough cognitive assessment to help clarify the clinical picture and guide your approach to the capacity assessment. The Montreal Cognitive Assessment (MoCA) and Addenbrooke's Cognitive Examination III (ACE-III) are superior to the MMSE as they have a higher sensitivity for frontal impairment. Consider a bedside frontal lobe battery or referral to neuropsychology for more detailed testing of executive function. Be mindful that relatively preserved language skills may present a superficial but false picture of intact cognition.
3. Gather collateral information and build up a longitudinal picture of Mr X's day-to-day functioning at home and on the ward. Involve multidisciplinary colleagues, including occupational therapist assessments, social care reports and nursing observations. This is all ‘relevant information’ that should be put to Mr X at interview.
4. Consider the specific capacity decision at hand. Guidance is available on relevant information that must be provided in assessments of capacity to consent to care and residence.^43^ This includes the areas Mr X needs support with, what would happen without support, and the sort of care he would receive in a particular placement (e.g. whether alcohol would be restricted).
5. Explore Mr X's own beliefs and values and how he might best be engaged and supported in his decision-making.^1^ Does he have family, friends or a trusted key worker who might help him to take information on board? Would he consider visiting a care home to get a concrete sense of what life might be like there?
6. Assess Mr X's capacity to make the decision using the MCA criteria, namely his ability to understand, retain and use or weigh relevant information, and to communicate his decision.^44^ Can he grasp the MDT's concerns about his cognition and care needs? Can he retain information about relevant past events for sufficient time to make a decision? Can he appreciate potential risks and weigh these in the balance? Rather than just ‘talk the talk’, can he use information at the time the decision needs to be made?^45^
7. Be mindful of the potential for reversibility of cognitive impairment and the need to schedule repeat capacity assessment(s) for Mr X after an appropriate interval.
8. Finally, although not the focus of this article, it is important to remember that even if Mr X lacks capacity regarding care decisions, the question of his best interests requires separate consideration under the MCA.

## Conclusions

Assessing capacity in people with ARBD can be clinically and ethically complex. Although alcohol dependence alone is generally not considered sufficient to impair capacity, it adds complexity in ARBD cases. Awareness of possible decision-making impairments and how these might interact with the decision at hand can provide helpful context in approaching individual capacity assessments for people with ARBD. The timing of the assessment is particularly important, given potential acuity and reversibility in this clinical presentation. Collateral information and multidisciplinary assessment can be key in establishing the relevant information that the individual must understand, retain, use and weigh. In terms of further research, clinical studies of capacity in people with ARBD (including those with active alcohol dependence) would be useful; this work could help clinicians approaching capacity assessments and developing decision-making supports.

## Data Availability

Data availability is not applicable to this article as no new data were created or analysed in this study.

## About the authors

**Nuala B. Kane** is a consultant general adult psychiatrist at Dublin Southeast Mental Health Service, Health Service Executive, Dublin, Ireland. **Norella Broderick** is a specialist registrar at Dublin Southeast Mental Health Service, Health Service Executive, Dublin, Ireland. **Emily Rao** is a medical student at the University of California San Diego School of Medicine, La Jolla, California, USA. **Alex Ruck Keene** is a barrister and Visiting Professor at the Dickson Poon School of Law, King's College London, London, UK. **Rahul Tony Rao** is a consultant old age psychiatrist and Visiting Clinical Research Fellow at the Institute of Psychiatry Psychology and Neuroscience, King's College London, London, UK.

## References

[1] Kane NB, Ruck Keene A, Owen GS, Kim SY. Difficult capacity cases – the experience of liaison psychiatrists. An interview study across three jurisdictions. Front Psychiatry 2022; 13: 946234. 35898632 10.3389/fpsyt.2022.946234 PMC9309683

[2] Hazelton LD, Sterns GL, Chisholm T. Decision-making capacity and alcohol abuse: clinical and ethical considerations in personal care choices. Gen Hosp Psychiatry 2003; 25: 130–5. 12676427 10.1016/s0163-8343(03)00005-7

[3] Perkins C, Hopkins J. Ethical issues associated with alcohol-related cognitive impairment. In Alcohol and the Adult Brain (eds J Svanberg, A Withall, B Draper, S Bowden): 192–205. Psychology Press, 2014.

[4] Rao R, Topiwala A. Alcohol use disorders and the brain. Addiction 2020; 115: 1580–9. 32112474 10.1111/add.15023

[5] Jauhar S, Smith ID. Alcohol-related brain damage: not a silent epidemic. Br J Psychiatry 2009; 194: 287–8. 10.1192/bjp.194.3.287b 19252162

[6] Rehm J, Hasan OS, Black SE, Shield KD, Schwarzinger M. Alcohol use and dementia: a systematic scoping review. Alzheimers Res Ther 2019; 11(1): 1. 30611304 10.1186/s13195-018-0453-0 PMC6320619

[7] Bernardin F, Maheut-Bosser A, Paille F. Cognitive impairments in alcohol-dependent subjects. Front Psychiatry 2014; 5: 78. 25076914 10.3389/fpsyt.2014.00078 PMC4099962

[8] Ridley NJ, Draper B, Withall A. Alcohol-related dementia: an update of the evidence. Alzheimers Res Ther 2013; 5(1): 3. 23347747 10.1186/alzrt157 PMC3580328

[9] Cheon Y, Park J, Joe K-H, Kim D-J. The effect of 12-week open-label memantine treatment on cognitive function improvement in patients with alcohol-related dementia. Int J Neuropsychopharmacol 2008; 11: 971–83. 18346293 10.1017/S1461145708008663

[10] Rustembegović A, Kundurović Z, Sapcanin A, Sofic E. A placebo-controlled study of memantine (Ebixa) in dementia of Wernicke-Korsakoff syndrome. Medicinski Arhiv 2003; 57: 149–50. 12858653

[11] Rao R. Outcomes from liaison psychiatry referrals for older people with alcohol use disorders in the UK. Ment Health Subst Use 2013; 6: 362–8.

[12] Bechara A, Noel X, Crone EA. Loss of willpower: abnormal neural mechanisms of impulse control and decision making in addiction. In Handbook of Implicit Cognition and Addiction (eds R. W. Wiers & A. W. Stacy): 215–32. Sage Publications, 2006.

[13] Noël X, Brevers D, Bechara A. A neurocognitive approach to understanding the neurobiology of addiction. Curr Opin Neurobiol 2013; 23: 632–8. 23395462 10.1016/j.conb.2013.01.018 PMC3670974

[14] Bickel WK, Miller ML, Yi R, Kowal BP, Lindquist DM, Pitcock JA. Behavioral and neuroeconomics of drug addiction: competing neural systems and temporal discounting processes. Drug Alcohol Depend 2007; 90(suppl 1): S85–91. 17101239 10.1016/j.drugalcdep.2006.09.016 PMC2033431

[15] Brevers D, Bechara A, Cleeremans A, Kornreich C, Verbanck P, Noël X. Impaired decision-making under risk in individuals with alcohol dependence. Alcoholism 2014; 38: 1924–31. 24948198 10.1111/acer.12447 PMC4115290

[16] Körner N, Schmidt P, Soyka M. Decision making and impulsiveness in abstinent alcohol-dependent people and healthy individuals: a neuropsychological examination. Subst Abuse Treat Prev Policy 2015; 10: 24. 26082020 10.1186/s13011-015-0020-7 PMC4477592

[17] Bowden-Jones H, McPhillips M, Rogers R, Hutton S, Joyce E. Risk-taking on tests sensitive to ventromedial prefrontal cortex dysfunction predicts early relapse in alcohol dependency: a pilot study. J Neuropsychiatry Clin Neurosci 2005; 17: 417–20. 16179667 10.1176/jnp.17.3.417

[18] Kovács I, Richman MJ, Janka Z, Maraz A, Andó B. Decision making measured by the Iowa gambling task in alcohol use disorder and gambling disorder: a systematic review and meta-analysis. Drug Alcohol Depend 2017; 181: 152–61. 29055269 10.1016/j.drugalcdep.2017.09.023

[19] Bechara A, Damasio AR, Damasio H, Anderson SW. Insensitivity to future consequences following damage to human prefrontal cortex. Cognition 1994; 50: 7–15. 8039375 10.1016/0010-0277(94)90018-3

[20] Boettger S, Bergman M, Jenewein J, Boettger S. Assessment of decisional capacity: prevalence of medical illness and psychiatric comorbidities. Palliative Support care 2015; 13: 1275–81. 10.1017/S1478951514001266 25355466

[21] Triebel K, Martin R, Novack T, Dreer L, Turner C, Pritchard P, et al. Treatment consent capacity in patients with traumatic brain injury across a range of injury severity. Neurology 2012; 78: 1472–8. 22496195 10.1212/WNL.0b013e3182553c38 PMC3345615

[22] Dreer LE, DeVivo MJ, Novack TA, Marson DC. Financial capacity following traumatic brain injury: a six-month longitudinal study. Rehabil Psychol 2012; 57: 5–12. 22369113 10.1037/a0025818 PMC4692242

[23] Kim SY, Karlawish JH, Caine ED. Current state of research on decision-making competence of cognitively impaired elderly persons. Am J Geriatr Psychiatry 2002; 10: 151–65. 11925276

[24] Marson DC, Hawkins L, McInturff B, Harrell LE. Cognitive models that predict physician judgments of capacity to consent in mild Alzheimer's disease. J Am Geriatr Soc 1997; 45: 458–64. 9100715 10.1111/j.1532-5415.1997.tb05171.x

[25] Martin RC, Triebel K, Dreer LE, Novack TA, Turner C, Marson DC. Neurocognitive predictors of financial capacity in traumatic brain injury. J Head Trauma Rehabil 2012; 27: E81–90. 23131973 10.1097/HTR.0b013e318273de49 PMC4709025

[26] Dreer LE, DeVivo MJ, Novack TA, Krzywanski S, Marson DC. Cognitive predictors of medical decision-making capacity in traumatic brain injury. Rehabil Psychol 2008; 53: 486–97. 20686627 10.1037/a0013798 PMC2914316

[27] Owen GS, Freyenhagen F, Martin W, David AS. Clinical assessment of decision-making capacity in acquired brain injury with personality change. Neuropsychol Rehabil 2017; 27: 133–48. 26088818 10.1080/09602011.2015.1053948 PMC5080972

[28] Mental Health and Justice Project. Neuroscience and Psychology in Capacity Assessments (Capacity Guide). Mental Health and Justice, 2022 (https://capacityguide.org.uk/neuroscience-and-psychology-in-capacity-assessments/).

[29] Ruck Keene A, Kane NB, Kim SY, Owen GS. Taking capacity seriously? Ten years of mental capacity disputes before England's court of protection. Int J Law Psychiatry 2019; 62: 56–76. 30616855 10.1016/j.ijlp.2018.11.005 PMC6338675

[30] McWilliams A, Fleming SM, David AS, Owen G. The use of neuroscience and psychological measurement in England's court of protection. Front Psychiatry 2020; 11: 570709. 33364988 10.3389/fpsyt.2020.570709 PMC7750429

[31] Craigie J, Davies A. Problems of control: alcohol dependence, anorexia nervosa, and the flexible interpretation of mental incapacity tests. Med Law Rev 2019; 27: 215–41. 30053254 10.1093/medlaw/fwy022 PMC6536256

[32] Charland LC. Decision-making capacity and responsibility in addiction. In Addiction and Responsibility (eds J Poland, G Graham): 139–58. MIT Press, 2011.

[33] Foddy B, Savulescu J. Addiction and autonomy: can addicted people consent to the prescription of their drug of addiction? Bioethics 2006; 20(1): 1–15. 16680876 10.1111/j.1467-8519.2006.00470.x

[34] Marshall KD, Derse AR, Weiner SG, Joseph JW. Revive and refuse: capacity, autonomy, and refusal of care after opioid overdose. Am J Bioethics [Epub ahead of print] 23 May 2023. Available from: 10.1080/15265161.2023.2209534. 37220012

[35] Pickard H. The purpose in chronic addiction. AJOB Neurosci 2012; 3: 40–9. 22724074 10.1080/21507740.2012.663058 PMC3378040

[36] Bandura A, Freeman WH, Lightsey R. Self-Efficacy: The Exercise of Control. Springer, 1999.

[37] Broadstock M, Brinson D, Weston A. The Effectiveness of Compulsory, Residential Treatment of Chronic Alcohol or Drug Addiction in Non-Offenders. Health Services Assessment Collaboration (HSAC) Report. University of Canterbury, 2008.

[38] Ministry of Health – Manatū Hauora (New Zealand). Substance Addiction (Compulsory Assessment and Treatment) Act 2017. New Zealand Government, 2017 (https://www.health.govt.nz/our-work/mental-health-and-addiction/mental-health-legislation/substance-addiction-compulsory-assessment-and-treatment-act-2017).

[39] Department of Health (Australia). Severe Substance Dependence Act. Victorian Government, 2010. (https://www.health.vic.gov.au/aod-policy-research-legislation/severe-substance-dependence-treatment-act).

[40] Nassif WM. Assessing decisional capacity in patients with substance use disorders. Curr Psychiatry 2019; 19: 35–40.

[41] Preston-Shoot M, Ward M. How to Use Legal Powers to Safeguard Highly Vulnerable Dependent Drinkers in England and Wales. Alcohol Change UK, 2021 (https://alcoholchange.org.uk/publication/how-to-use-legal-powers-to-safeguard-highly-vulnerable-dependent-drinkers).

[42] Galvin R, Bråthen G, Ivashynka A, Hillbom M, Tanasescu R, Leone M. EFNS guidelines for diagnosis, therapy and prevention of Wernicke encephalopathy. Eur J Neurol 2010; 17: 1408–18. 20642790 10.1111/j.1468-1331.2010.03153.x

[43] 39 Essex Chambers. Guidance Note: Relevant Information for Different Categories of Decisions. 39 Essex Chambers, 2021 (https://www.39essex.com/sites/default/files/Mental-Capacity-Guidance-Note-Relevant-Information-for-Different-Categories-of-Decision-1.pdf).

[44] Mental Health and Justice Project. Understanding the Functional Criteria (Capacity Guide). Mental Health and Justice, 2022 (https://capacityguide.org.uk/practical-legal-guidelines/understanding-the-functional-criteria/).

[45] The Mental Health and Justice Project. The Quality of Lack of ‘Online Awareness’ of Deficit (Capacity Guide). Mental Health and Justice, 2022 (https://capacityguide.org.uk/the-quality-of-lack-of-online-awareness-of-deficit/).

